# Supplementary figures and images for: Cellular Immune Profiling of Lung and Blood Compartments in Patients with SARS-CoV-2 Infection
Source: Pathogens. 2023 Mar 11;12(3):442. doi: 10.3390/pathogens12030442 (PMC10057444; doi:10.3390/pathogens12030442)

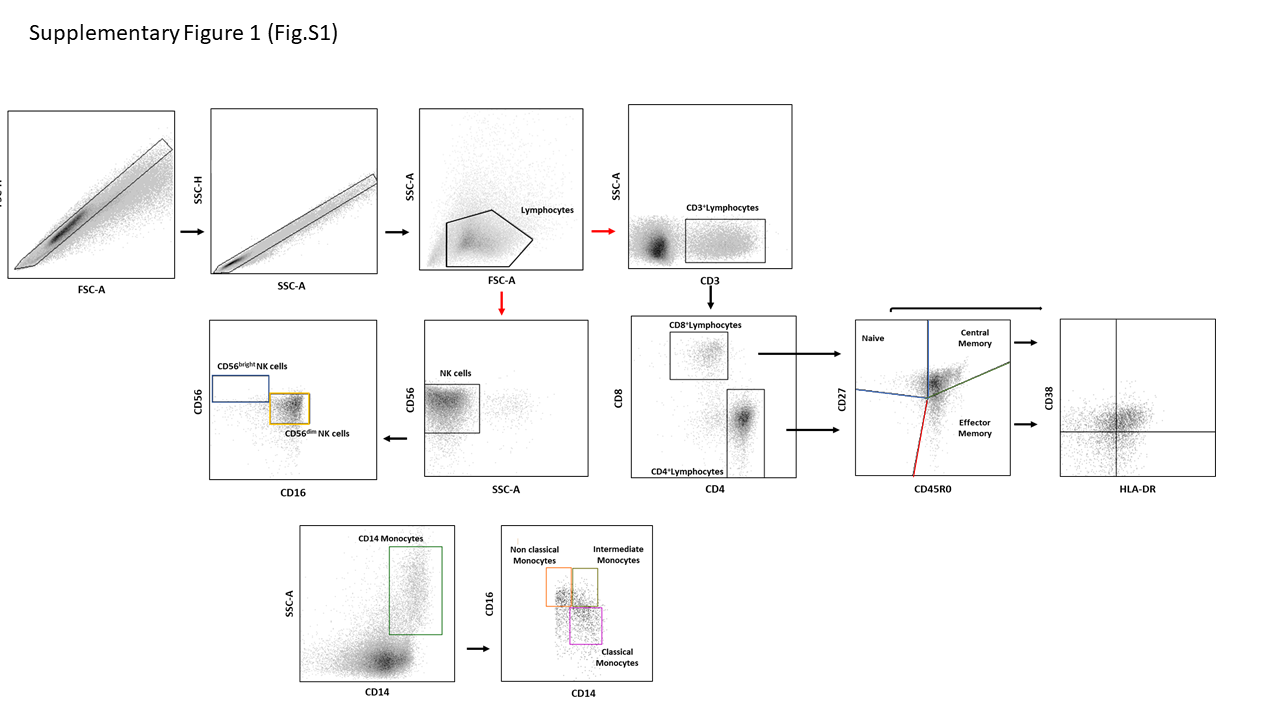

Supplement: Supplementary file 1 [file pathogens-12-00442-s001.zip › pathogens-2214969-supplementary.tif]
